# Social Jet Lag Associates Negatively with the Adherence to the Mediterranean Diet and Body Mass Index among Young Adults

**DOI:** 10.3390/nu11081756

**Published:** 2019-07-30

**Authors:** María Fernanda Zerón-Rugerio, Trinitat Cambras, Maria Izquierdo-Pulido

**Affiliations:** 1 Department of Nutrition, Food Science and Gastronomy, School of Pharmacy and Food Science, University of Barcelona, 08028 Barcelona, Spain; 2 INSA-UB, Nutrition and Food Safety Research Institute, University of Barcelona, 08921 Santa Coloma de Gramenet, Spain; 3 Department of Biochemistry and Physiology, School of Pharmacy and Food Science, University of Barcelona, 08028 Barcelona, Spain; 4 CIBER Physiopathology of Obesity and Nutrition (CIBEROBN), Institute of Health Carlos III, 28029 Madrid, Spain

**Keywords:** social jet lag, BMI, Mediterranean diet, chronotype, young adults

## Abstract

Obesity and unhealthy eating habits have been associated with irregular sleep–wake patterns during the week, also known as social jet lag. The Mediterranean diet is a healthy pattern related with a better health and sleep quality. However, potential associations with social jet lag remain unexplored. The aim of this study was to examine whether higher social jet lag is linked to lower adherence to the Mediterranean diet and whether it is associated with BMI (Body Mass Index). This cross-sectional study included 534 young adults (18–25 years). Anthropometric parameters, adherence to the Mediterranean diet, chronotype and social jet lag were studied. Our results revealed that individuals with greater social jet lag showed lower adherence to the Mediterranean diet and had a higher BMI. Among the habits that characterized the Mediterranean dietary pattern, we observed that higher social jet lag was significantly associated with a lower intake of fruits and vegetables, as well as skipping breakfast. Hence, the promotion of regular sleep habits together with healthy dietary patterns should be considered for obesity prevention, especially among young adults.

## 1. Introduction

Young adulthood is a period marked by important transitions, such as leaving home and an increase of autonomous decision making. This period may be an important, yet overlooked, stage of life for establishing long-term health behavior patterns, including healthy dietary, sleep and physical activity patterns [1]. Research from longitudinal studies has identified the transition between adolescence and adulthood as a period of increased risk for excess weight gain, decrease in diet quality and in physical activity [1]. In addition, the modernization of society has brought certain sociological changes, such as sedentary lifestyles, long store hours, late-night sports events, energy drinks, less time for food preparation and changes in eating patterns (e.g., an increased intake of snacks, more frequent breakfast skipping and decreasing intake of fruits and vegetables) [2,3,4,5].

Additionally, a rise in the Western diet (characterized by the presence of foods with high quantities of sugar, salt and saturated fats) has been observed among the young population [6]. In most Mediterranean countries (including Spain), the Mediterranean dietary pattern is being abandoned by young generations. Taking note, the Mediterranean diet is based on a high consumption of vegetables and fruits, cereals and whole grains, pulses and nuts, moderate to high intake of fish and seafood, olive oil as the main (added) fat, and low to moderate intake of dairy products [7,8,9]. Interestingly, despite its high fat content, emerging evidence indicates that the Mediterranean diet can counterbalance the detrimental effects of central obesity associated with chronic low-grade inflammation [7]. In this context, the Mediterranean diet is recognized as a healthy eating pattern which contributes to better health and quality of life. 

Besides changes in food consumption patterns, the exposure to artificial light at night and the use of electronic devices 24/7 are common in contemporary society [3]. The later may delay sleep onset and thus disrupt circadian rhythms, emerging as a potential risk factor for obesity [10]. Importantly, Chaput et al. pointed out that people living in a 24/7 society place sleep down on their priority list [3]. Even “usual” working hours can result in a subtle kind of circadian misalignment due to sleep deprivation during workdays, especially among individuals who prefer late bedtimes and later awakening (*evening chronotypes*) [11]. In addition, most school and work schedules are morning oriented, forcing evening chronotypes to use alarm clocks to align their wake times with social obligations [12]. Hence, sleep is curtailed, and individuals are prone to a mild kind of misalignment (*social jet lag*) [13] between their internal circadian clock and actual sleep–wake times, with a potential impact on metabolic health [11]. Moreover, because sleep is regulated by both circadian and homeostatic processes, sleep duration tends to be longer during weekends as an attempt to compensate the sleep debt accumulated through the week [3,11,14]. 

Social jet lag, given by the difference between the midpoint of sleep on weekends and workdays, was first described by Wittman et al. [15] as a measure of circadian misalignment. Since the beginning, social jet lag was linked to low sleep quality, eveningness, higher exposure to artificial light at night, and higher caffeine consumption. In addition, Wittman et al. pointed out that adolescence and young adulthood was a potential risk factor for social jet lag [15]. It is important to highlight that these are stages of life in which diurnal preference is delayed, reaching its maximum around the age of 20 [16]. Subsequent studies have demonstrated that social jet lag is also associated with obesity and metabolic disorders in both adolescents and adults [11,12,17,18,19]. Among several potential mechanisms, circadian misalignment and sleep restriction seem to be important factors leading to adverse effects on metabolism (especially disruption in glucose metabolism) and weight-gain [10,12]. In addition, recent studies have shown that social jet lag is associated with an increased intake of total fat and saturated fat [20], higher consumption of sweets, and unhealthy dietary habits, including increased snacking and longer eating duration [20].

However, to our knowledge, social jet lag has not yet been related to the adherence to a healthy dietary pattern, such as the Mediterranean diet. Additionally, there are several advantages supporting the study of dietary patterns versus single nutrients in health promotion. Thus, our aim was to explore the potential association between social jet lag and adherence to the Mediterranean diet in a population of Spanish young adults, who are culturally attached to this dietary pattern. We hypothesized that individuals with greater social jet lag will have less adherence to the Mediterranean diet and higher BMI. 

## 2. Materials and Methods

### 2.1. Study Design, Settings, Participants and Protocol

Subjects aged 18–25 years were recruited in a cross-sectional study during the school year, among undergraduate and postgraduate students at the University of Barcelona (Spain), between 2017 and 2019. Recruitment consisted of an informative talk, explaining details to the students about the research, and encouraging them to take part in the study. Eligibility criteria included undergraduate and postgraduate students enrolled in the University of Barcelona and being between 18 and 25 years old. Exclusion criteria included failing to provide information necessary for the development of the study or being previously diagnosed with chronic diseases such as type 2 diabetes, hypertension and cardiovascular disease. Participants completed the online questionnaire using Survey CTO [21], a web interface which allowed us to collect and securely manage the data, thanks to multiple layers of encryption. Of note, subjects had the option to leave the study at any time. From the 559 subjects included in the study, 25 subjects were excluded for not meeting the eligibility criteria; therefore, the sample finally included 534 subjects for the analysis. The study was approved by the Ethics Committee of the University of Barcelona and all the participants gave written informed consent according to the general recommendations of the Declaration of Helsinki. 

### 2.2. Data Collection

We used Open Data Kit (ODK) [22], which is an open-source software to design an online screening tool, where we asked standard questions (described below) including anthropometric parameters, adherence to the Mediterranean diet, sleep duration and timing, chronotype and physical activity. ODK has a user-friendly web interface for designing web forms and programming simple logic. 

### 2.3. Anthropometric Parameters

Self-reported height and weight were queried in a questionnaire as ‘What is your current weight? (in kg)’ and ‘What is your current height? (in cm)’. Self-reported BMI was calculated from these values. Furthermore, to validate self-measured BMI, a randomly selected subset (n = 200) that was stratified according to participants’ age and gender was drawn to objectively evaluate weight and height [23]. Subjects were weighed wearing light clothes and without shoes to the nearest 0.1 kg (Seca 703 scale, Seca, Hamburg, Germany), height was determined using a fixed wall stadiometer (Seca 217, Seca) to the nearest 0.1 cm, and BMI was calculated and compared to self-reported BMI. 

### 2.4. Adherence to the Mediterranean Diet

The Mediterranean Diet Quality Index for children and adolescents (KIDMED) was used to evaluate adherence to the Mediterranean diet in the Spanish young population [2]. The KIDMED test is based on the principles that sustain Mediterranean dietary patterns and those that undermine it. Briefly, the KIDMED test includes questions like: ‘Do you have fruit or fruit juice every day?’, ‘Do you have a second piece of fruit every day?’, ‘Do you have fresh or cooked vegetables regularly once a day?’, ‘Do you have fresh or cooked vegetables more than once a day?’, ‘Do you consume nuts regularly (at least 2–3 times per week)?’, and ‘Do you go more than once a week to a fast-food (hamburger, pizza) restaurant?’. Items denoting lower adherence to the Mediterranean diet were assigned a value of −1 and those related to higher adherence were scored +1. Scores range from −4 to 12, with higher scores indicating greater adherence to the Mediterranean Diet. In addition, according to the score, adherence can be characterized as poor (δ3), average (4–7), or good (ε8). 

### 2.5. Sleep Duration

Habitual sleep duration was estimated by a questionnaire including the questions ‘During weekdays/weekends: At what time do you usually go to bed?’, ‘During weekdays/weekends: At what time do you usually wake up?’. A total weekly duration was calculated as: [5 × weekday sleep (hr) + 2 × weekend sleep (hr)]/7 [24]. 

### 2.6. Social Jet Lag

Social jet lag, the discrepancy between the internal and external timing, was measured by subtracting each participant’s midpoint between bedtime and wake up time (midpoint of sleep) on workdays, from the midpoint of sleep on weekends [15]. All analyses were conducted using the absolute value of social jet lag [15,17]. 

### 2.7. Chronotype

Chronotype (morning–eveningness typology) is a way to characterize subjects depending on individual preferences of wake/sleep patterns and the time of the day people report to perform best. Some people are ‘night owls’ and like to stay late in the night and sleep late in the morning (evening types), whereas others are ‘early birds’ and prefer to go to bed early and arise with the break of dawn (morning types). To evaluate chronotype, subjects completed the 19-item Morningness and Eveningness Questionnaire (MEQ) (Score range: 10–86) of Horne and Ostberg [25]. According to the score, individuals are categorized as intermediate (42–58), morning (>58) or evening (<42) types. 

### 2.8. Physical Activity

Physical activity was assessed using the short version of the International Physical Activity Questionnaire (IPAQ) [26]. This version of the IPAQ questionnaire has been validated in the Spanish population, in which a good correlation with accelerometer data was obtained. This screening tool contains questions like: ‘During the last 7 days, on how many days did you do vigorous physical activities like heavy lifting, digging, aerobics or fast bicycling?’, ‘How much time did you usually spend doing vigorous physical activities on one of those days’, ‘During the last 7 days, on how many days did you do moderate physical activities like carrying light loads, bicycling at a regular pace, or doubles tennis? Do not include walking’, ‘How much time did you usually spend doing moderate physical activities on one of those days’, ‘During the last 7 days, on how many days did you walk for at least 10 minutes at a time?’, and ‘How much time did you usually spend walking on one of those days?’. Physical activity score was calculated based upon the frequency (in days), duration (in minutes) and the Metabolic Equivalent of Task (MET) according to each type of physical activity (8 METs for vigorous activity, 4 METs for moderate and 3.3 METs for walking). Total physical activity score was calculated adding the three IPAQ domains [26].

### 2.9. Statistical Analyses

Descriptive characteristics are presented for all participants, including means and standard deviations for continuous variables (age, BMI, physical activity, KIDMED score, chronotype score, sleep variables and social jetlag) and percentages for categorical variables (sex, BMI, KIDMED Index and chronotype). Pearson correlations were used to investigate associations between circadian and sleep related variables with adherence to the Mediterranean diet and BMI. Any significant relationship between variables was further investigated with regression analyses and adjusted by confounding variables. Student’s t-test was used to compare crude means of social jet lag and the KIDMED test items. Further statistical adjustment was performed for gender, age and physical activity (analysis of covariance). *p*-values were corrected using the Benjamini–Hochberg method, assuming a False Discovery Rate (FDR) of 5%. Paired t-tests were used to compare the means between sleep duration on weekends and workdays and self-reported BMI with nutritionist-measured BMI. Further analyses using linear regression model was performed to test the association between self-reported measures and objective measurements. All analyses were performed with the SPSS statistical computer software, version 24.0 (IBM SPSS Statistics), except for FDR which was performed using R Software. Significance testing was considered when *p* < 0.05.

## 3. Results

### 3.1. Participants’ Characteristics, Social Jet Lag and Adherence to the Mediterranean Diet

The majority of the studied population showed a normal weight, while 12.6% were overweight or obese (Table 1). The validation of self-reported BMI showed that self-reported BMI was on average similar to measured BMI (r^2^ = 0.915; β = 0.921; 95% CI [0.882, 0.961]; *p* < 0.001). The mean difference between self-measurement and measurement was −0.23 (0.99) kg/m^2^. These results implied that they could be used simultaneously without any correction coefficient in the main analyses of this study. Regarding physical activity, the studied young population showed a moderate level of physical activity. The sleep duration was significantly shorter on workdays (7.8 ± 0.9 hours) than on weekends (9.0 ± 0.9 hours) (*p* < 0.001). Interestingly, we observed that the 77% of the studied population showed more than 1 hour of social jet lag, out of which 33% had more than 2 hours of social jet lag. 

Table 2 shows the adherence to the Mediterranean diet score and the frequency of consumption by food groups and habits that are part of this dietary pattern. More than half of the studied population had a poor/average adherence to the Mediterranean diet. We observed that only one- third of the participants had a second serving of fruit and vegetables, and that less than half of the studied population consumes nuts more than 2–3 times a week. 

### 3.2. Higher Social Jet Lag Is Associated with a Lower Adherence to the Mediterranean Diet and Higher BMI

As shown in Table 3, our results proved that subjects with higher values of social jet lag showed a lower adherence to Mediterranean diet and had higher BMI. In addition, evening-type subjects showed higher BMI, while a moderate trend toward significance was observed between eveningness and lower adherence to the Mediterranean diet (*p* = 0.057). 

### 3.3. Social Jet Lag Is Associated with Lower Intake of Fruits, Vegetables and Breakfast Skipping

**Figure 1:**
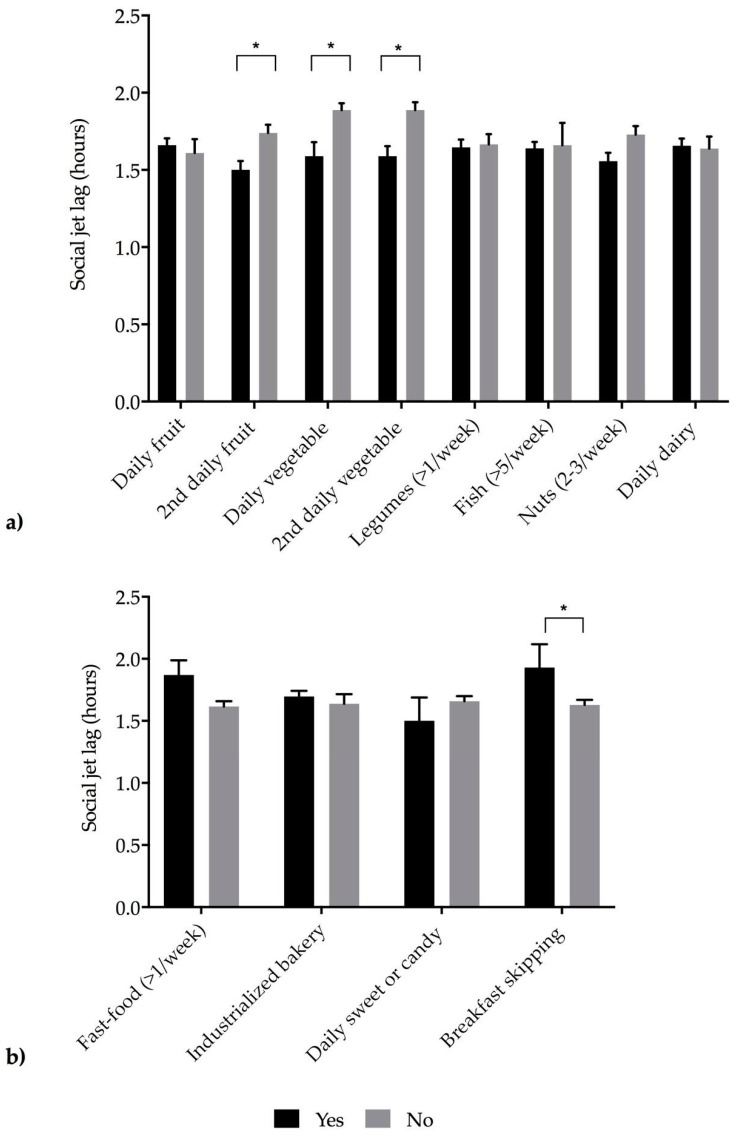
Social jet lag (in hours) and accomplishment or not of the KIDMED items. KIDMED, Mediterranean Diet Quality Index. Figure (**a**) shows healthy food groups, and (**b**) unhealthy dietary habits. Bars indicate mean values (standard deviations). Black bars represent the consumption of food group or dietary habits, and grey solid bars show lack of consumption of a food group or dietary habit. Data were analyzed using ANCOVA tests, adjusted by the following covariates: age, gender and physical activity. Significant *p*-values * < 0.05.

As it is shown in Figure 1a, individuals who reported not consuming a second serving of fruit, not consuming fresh or cooked vegetables regularly (once a day) or not consuming fresh or cooked vegetables more than once a day, showed higher values of social jet lag (*p* < 0.05). Interestingly, subjects who skipped breakfast also presented higher social jet lag (*p* < 0.05) (Figure 1b). 

## 4. Discussion

To our knowledge, this is the first cross-sectional study to demonstrate that higher social jet lag is associated with lower adherence to the Mediterranean diet in a population of young adults. Interestingly, we observed that a higher BMI was associated with higher social jet lag. These associations remained significant even after adjusting for confounding variables. As far as we are aware, only one study has investigated the relationship between social jet lag and healthy dietary patterns, and this was in a cohort of British adults (aged 19–64 years) [27]. In consonance with our results, the authors found that lower adherence to a healthy pattern (which included consumption of fruits, vegetables, yogurts, low fat cheese and dairy, oily-fish, high-fiber breakfast cereals, nuts and seeds) was associated with a higher social jet lag. However, unlike our study, the healthy dietary pattern score was derived from principal component analysis and social jet lag was calculated using the difference between sleep duration on weekends and workdays. In any case, it is important that similar results account for two populations with different eating habits and schedules.

Regarding the habits that characterized the Mediterranean dietary pattern and its interaction with social jet lag, we observed that individuals with greater social jet lag often missed their daily vegetable serving, as well as their second daily servings of fruit and/or vegetables. To our knowledge, only few studies have studied the association between social jet lag and food intake. A cross-sectional study conducted with a cohort of Brazilian undergraduate students pointed out that social jet lag was negatively associated with bean intake, which is a typical constituent of the Brazilian diet considered protective against weight-gain [28]. In the same line, Mota et al. [20] recently showed that having more than 1 hour of social jet lag was associated with a higher intake of meat, eggs and sweets in a cohort of individuals with obesity-related chronic diseases, suggesting that social jet lag is associated with an unhealthy diet [20]. 

Among other unhealthy habits screened by the KIDMED test, we observed that subjects with greater social jet lag often skipped breakfast. It is worth mentioning that the habit of eating breakfast is considered an important indicator of health, and that overweight or obesity have been highly associated with breakfast skipping [29,30]. A cross-sectional study conducted with Brazilian undergraduate students pointed out that social jet lag was higher in evening-type subjects and the frequency of breakfast skipping within these participants was high [29]. Interestingly, Teixeira et al. [29] pointed out that students who skipped breakfast were 2.3 times more likely to be overweight when compared to non-breakfast skippers. Moreover, a recent study suggested that regular breakfast consumption is an important habit towards weight-maintenance [30]. So, breakfast skipping may be a contributing factor in the association between greater social jet lag and increased BMI in our sample of young adults. 

Although the mechanisms linking low-diet quality and social jet lag remain relatively unexplored, we hypothesize that the variability in sleep duration between workdays and weekends may be another plausible explanation underlying these associations. A prospective study performed in a large cohort of Japanese healthy adults, reported variability of sleep duration linked to weight gain over a 3-year period in healthy adults [31]. The authors postulated that irregular sleep patterns may lead to arrhythmic exposure to light and constant resetting of the central oscillator. This, together with, extended periods of wakefulness and fragmented sleep schedules may in turn alter normal feeding patterns and desynchronize peripheral oscillators in metabolic tissues. Other studies reported that the presence of high variance in sleep duration was associated with adverse metabolic outcomes (altered insulin, low-density lipoprotein and high-sensitivity C-reactive protein plasma levels) and abdominal obesity in children and adolescents [32,33]. 

Additionally, few studies have reported inverse associations between poor sleep quality and adherence to the Mediterranean diet in adolescents and adults [34,35,36]. Ferranti et al. [34] highlighted that Italian adolescents (aged 11–14 years) who habitually slept less (8.5 + 1.2 vs. 9.0 + 1.3 hours) were more likely to consume less vegetables and fruits and ate out more frequently [34]. Consistent with this finding, two other cross-sectional studies pointed out that short sleep duration was associated with poor diet quality, assessed with the Healthy Eating Index [37]. Among the main mechanisms, several authors have postulated that sleep restriction increases food intake via (i) changes in appetite-related hormones (mainly ghrelin and leptin), (ii) enhanced neuronal activity in response to food stimuli, increasing food intake via hedonic mechanisms, (iii) providing extended hours of wakefulness, presenting additional opportunities to eat, and (iv) delaying meal timing, which has been suggested as an independent predictor of BMI [37,38,39]. 

As mentioned above, BMI was associated with higher social jet lag even though only a small percentage of our population was overweight or obese. This relationship has been previously described in general population (adolescents and adults) [12,17,19,40]; however, to our knowledge this is the first time that it has been reported in a population of young adults. Different reasons may be driving this association, such as unhealthy dietary habits and poor dietary intake, which has been addressed above. Additionally, alterations in glucose metabolism caused by chronic circadian disruption may be a relevant mechanism underlying obesity and social jet lag [17,41,42]. Shorter sleep duration is associated with a shift in the melatonin rhythm, resulting in high melatonin levels in the morning upon awakening and eating the morning meal during the biological night, which reflects misalignment of the central and peripheral clocks [13]. It is important to highlight that social jet lag is a condition that occurs chronically from adolescence and it is usually maintained through the individual’s working life [12]. This emphasizes social jet lag as a potential risk factor for obesity, and also as a key factor triggering the development of several chronic conditions (e.g., metabolic syndrome or type 2 Diabetes). 

Being an owl, as evening-type subjects are commonly called, is another risk factor for social jet lag and obesity among adolescents and adults [43,44,45]. Agreeing with that, we found a significant relationship between eveningness and higher BMI. Several authors pointed out that circadian misalignment, as well as delaying meal timing and sleep onset, are the main mechanisms supporting this association [12,24,28,44]. Additionally, this preference towards later time-of-day is linked with unhealthy dietary habits, including higher intake of energy, sucrose, and fat compared with morning-types. Eveningness has also been related with lower adherence to the Baltic Sea Diet, a dietary pattern followed in Nordic countries, which includes berries, roots and cabbages, rye, oats and barley, low-fat milk products and fish [46,47]. Interestingly, we observed that being an owl was associated with lower adherence to the Mediterranean diet, although after adjustment this association was only close to statistical significance (*p* = 0.057).

Our study has certain limitations, starting with its cross-sectional nature, which prevented us from finding causation, the use of self-reported weight and height, and a questionnaire to assess adherence to the Mediterranean diet. Furthermore, sleep habits (bedtime and wakeup timing) were self-reported, and thus, sleep latency has not been considered. To help avoiding underreport in future studies, we suggest the use of objective data (such as actigraphy) which would be appropriate to verify circadian preferences or social jet lag. However, our sample size is large enough to provide sufficient strength to the associations between social jet lag, adherence to the Mediterranean diet and BMI. 

## 5. Conclusions

In this study, we showed that social jet lag is negatively associated with adherence to the Mediterranean diet and is positively associated with BMI in a sample of young adults. Our findings raise interesting questions and novel opportunities for obesity prevention among young adults. To this end, we consider that promoting sleep hygiene among young adults is highly relevant, especially since emerging adulthood is a stage of life accompanied by several life changes and where risk for obesity and chronic diseases may be established. Furthermore, regularity in sleep habits is as crucial as exercise and eating for overall health, so we hope more attention will be brought to sleep in the future. Therefore, we suggest that nutritional interventions should include sleep hygiene as an important strategy to improve dietary habits as well as BMI. This approach resonates with growing interest in personalized preventive care, especially since obesity is the result of the interaction of the individual with an obesogenic environment.

## Figures and Tables

**Table 1:** General characteristics of the population studied.

**Total Sample (n)**	534
**Gender, %F**	74.3
**Age, years**	21.5 (3.0)
**BMI, kg/m^2^**	21.7 (3.1)
Underweight, % (n)	10.1 (54)
Normal weight, % (n)	77.3 (413)
Overweight, % (n)	10.5 (56)
Obese, % (n)	2.1 (11)
**Physical Activity (METs)**	2353.9 (2701.5)
**Sleep related variables**	
Workdays	
Sleep onset, hh:mm	23:51 (00:49)
Sleep offset, hh:mm	07:37 (00:58)
Midpoint of sleep, hh:mm	03:45 (00:46)
Sleep duration, hours	7.8 (0.9)
Weekends	
Sleep onset, hh:mm	00:54 (01:09)
Sleep offset, hh:mm	09:52 (01:14)
Midpoint of sleep, hh:mm	05:23 (01:04)
Sleep duration, hours	9.0 (0.9)
**Social jet lag, hours**	1.6 (0.9)
**Chronotype**	49.0 (9.3)
Evening type, % (n)	20.6 (110)
Intermediate type, % (n)	62.4 (333)
Morning type, % (n)	17.0 (91)

BMI, body mass index; F, females; hh:mm, hours:minutes, METs, metabolic equivalent. Values are means (standard deviations) for continuous data and proportions (%) for categorical data.

**Table 2:** KIDMED Score, index, and frequencies of response to each item of the test.

	Population Studied (n = 534)
**KIDMED test score total, mean (SD)**	7.0 (2.1)
**KIDMED index, %**	
Poor (≤3 points)	4.9
Average (4–7 points)	50.7
Good (8–12 points)	44.4
**KIDMED test (%, yes)**	
Takes a fruit of fruit juice every day	78.1
Has a second serving of fruit every day	38.6
Has fresh or cooked vegetables regularly once a day	78.8
Has fresh or cooked vegetables more than once a day	40.3
Consumes fish regularly (at least 2–3 days/week)	61.8
Goes more than once a week to a fast-food (hamburger) restaurant	13.9
Likes pulses and eats them more than once a week	72.7
Consumes pasta or rice almost every day (≥5 times/week)	43.1
Has cereals or cereal products (bread) for breakfast	89.1
Consumes nuts regularly (at least 2–3 times per week)	44.6
Uses of olive oil at home	97.9
Skips breakfast	7.7
Has a dairy product for breakfast (yogurt, milk, etc.)	72.5
Takes two yogurts and/or some cheese (40 g) daily	40.1
Has commercially baked goods or pastries for breakfast	23.0
Takes sweets and candy several times every day	4.5

KIDMED, Mediterranean Diet Quality Index for children and adolescents. Values are means (standard deviations) for continuous data and proportions (%) for categorical data.

**Table 3:** Associations between adherence to Mediterranean diet, BMI and circadian related variables.

	Adherence to Mediterranean Diet			BMI		
	β	95% CI	*p* ^a^	β	95% CI	*p* ^a^
Social jet lag	−0.305	−0.503, −0.107	0.003	0.304	0.021, 0.587	0.035
Chronotype	0.019	−0.001, 0.038	0.057	−0.028	−0.055, −0.001	0.043

BMI, body mass index; CI, confidence interval. Data was analyzed using linear regression models to test associations with continuous outcome measures of adherence to the Mediterranean diet and BMI. Lower values of KIDMED indicate lower adherence to the Mediterranean diet, whereas lower MEQ values are associated with the evening chronotype. The table shows the unstandardized coefficient (β), CI and *p*-value associated with each predictor variable. ^a^ Adjusted by age, gender, physical activity and sleep duration.
